# Comparative analysis of biofilm formation by *Candida albicans* and *Candida krusei* in different types of contact lenses

**DOI:** 10.5935/0004-2749.20220033

**Published:** 2022

**Authors:** Laura Nagy Fritsch, Amanda Latercia Tranches Dias, Naiara Chaves Silva, Geraldo José Medeiros Fernandes, Flávia Beatriz de Andrade Oliveira Ribeiro

**Affiliations:** 1 Faculdade de Medicina, Universidade Federal de Alfenas, Alfenas, MG, Brazil; 2 Instituto de Ciências Biomédicas, Universidade Federal de Alfenas, Alfenas, MG, Brazil; 3 Programa de Pós-Graduação em Ciências Farmacêuticas, Universidade Federal de Alfenas, Alfenas, MG, Brazil; 4 Departamento de Anatomia, Faculdade de Medicina, Universidade José do Rosário Vellano, Alfenas, MG, Brazil; 5 Departamento de Oftalmologia, Faculdade de Medicina, Universidade Federal de Alfenas, Alfenas, MG, Brazil

**Keywords:** Biofilm, Contact lense, Contact lense, hydrophilic, *Candida albicans*, *Candida krusei*, Biofilme, Lente de contato, Lente de contato hidrofílica, *Candida albicans*, *Candida krusei*

## Abstract

**Objective:**

To evaluate the *Candida krusei* and *Candida albicans* biofilm formation abilities on 5 different types of contact lenses and compare their metabolic activities and biomass.

**Methods:**

After biofilm formation by both the test species, their metabolic activity was assessed by the 2,3-bis (2-methoxy-4-nitro-5-sulfophenyl)-2H-tetrazolium-5-carboxanilide reduction assay with menadione, while the biomass was determined by staining with 0.4% crystal violet dye for further statistical analysis.

**Results:**

Both the *Candida* species could form biofilms on different types of contact lenses, with greater metabolic activities and lower biomass formation in rigid gas permeable lenses.

**Conclusion:**

Biofilm formation with greater metabolic activity and greater biomass were expected on soft contact lenses considering their surface hydrophobicity. However, the results demonstrated a greater metabolic activity on rigid contact lenses. This result has a great significance with regards to the increasing risk of microbial keratitis, although further studies are warranted to better elucidate the formation of biofilms on different types of contact lens materials in the future.

## Abstract (pt)

**Objetivo:**

Avaliar a capacidade de formação de bio filmes de *Candida krusei* e *Candida albicans* em cinco tipos de lentes de contato, comparando atividade metabólica e biomassa dos mesmos.

**Métodos:**

Após a formação de biofilme de ambas as espécies, a atividade metabólica foi avaliada por ensaio de redução 2,3-bis (2-methoxy-4-nitro-5-sulfophenyl)-2H-tetra zolium-5-carboxanilide com Menadiona, e a biomassa foi avaliada por coloração com Cristal Violeta 0,4% para posterior análise estatística.

**Resultados:**

Ambas as espécies de *Candida* foram capazes de formar biofilmes nos diferentes tipos de lentes de contato, havendo em lentes rígidas gás permeável maior atividade metabólica e menor biomassa formada.

**Conclusão:**

Esperava-se a obtenção de biofilmes de maior atividade metabólica e maior biomassa em lentes de contato gelatinosas com base no fundamento da Hidrofobicidade Superficial. Porém, o resultado apontou para maior atividade metabólica em lentes de contato rígidas. Apesar de observados resultados significativos, trata-se de um assunto de grande importância frente ao aumento do número de ceratites microbianas, mostrando-se necessários outros estudos para melhor elucidar a formação de biofilmes em diferentes tipos de materiais de lentes de contato.

## INTRODUCTION

The popularization of the use of contact lenses runs parallel with reports of increased risk of microbial keratitis. Although fungal keratitis represents only 1.5% of all cases of keratitis in contact lenses users^(1)^, the presence of fungi and the consequent formation of biofilms in contact lenses is a growing threat to the public health^(2)^.

Most *Candida* spp. are biofilm producers on a large or small scale, which is an important factor associated with their virulence and resistance to antifungals, which in turn favor the occurrence of serious or recurrent infections^(3)^. Owing to the physical barrier of a polymeric matrix in relation to fungal biofilms, less susceptibility is associated with the penetration of antimicrobials of multipurpose solutions for the maintenance of contact lenses^(4)^.

The inappropriate handling of contact lenses by their users generates friction between the lenses and the cornea, causing reactions that ease the entry of infectious agents onto the lenses, although the mentioned organisms do not penetrate the whole cornea^(5)^.

According to the literature, the risk of complications from the use of soft contact lens subtypes is greater than that from the use of rigid ones^(6)^.

Concerning rigid gas permeable contact lenses, past data demonstrate a 21-times greater risk of microbial keratitis for programmed-replacement disposal lenses and a 4-times greater risk among daily-use contact lenses. In contrast, the analyses of only rigid contact lenses have shown only a slightly higher risk with polymethylmethacrylate contact lenses when compared to rigid gas permeable ones^(7)^.

Through this study, we aimed to broaden the considerably scarce knowledge database on the possibility of different types of contact lenses materials allowing the formation of biofilms formation by *C. albicans* and *C. krusei* toward the development of preventive and/or reductive measures against eye infections among contact lenses users.

## METHODS

The present study was conducted at the Microbiology and Immunology Laboratories from the Federal University of Alfenas (UNIFAL-MG). Two strains of *Candida* spp. were used, namely, *C. albicans* SC5314 and *C. krusei* ATCC^®^ 6258.

To assess and compare the capacity of biofilm formation in different types of contact lenses by the selected strains of *Candida* spp., 5 types of contact lenses were used in this study. Among the soft contact lenses, biofilms were formed in programmed-replacement disposal, single-use (daily disposable), and therapeutic contact lenses. Among rigid gas permeable contact lenses, the biofilms were formed in polycarbonate lenses and in Hexafocon A copolymer (XO^®^, Bausch and Lomb).

Biofilms were developed as suggested in the literature^(8)^ with some modifications^(9)^. Briefly, pre-sterilized commercial flat-bottom polystyrene 24-wells microplates with a 2-mL total well volume that could perfectly shelter the contact lenses were used in this study. *Candida* spp. was first cultured on Sabouraud’s Dextrose Agar medium and then in RPMI-1640 broth, using the 24-h incubation time at 37°C. The cell concentration was adjusted to 1-5 **×** 10^7^ cells/mL in RPMI-1640 broth by measuring the optical density (OD), which was nearly 0.4 at 530 nm. On the contact lenses, the culture suspensions were added and maintained for 2 h at 37°C on a shaker at 75 rpm to ensure cell adhesion. The plate/ lens sets were washed with PBS solution, non-inoculated RPMI was added, and the microplates were incubated again for 24 h at 37°C on 75-rpm rotation shaker for biofilm formation/development. The tests were performed thrice on different types of contact lenses.

The metabolic activities of biofilms were assessed by the 2,3-bis (2-methoxy-4-nitro-5-sulfophenyl)-2H-tetrazolium-5-carboxanilide (XTT) reduction assay as described elsewhere^(8)^ with some modifications^(9)^. The reagent solution was prepared at the ratio of 5:1 by mixing a 1 mg/mL XTT solution in PBS and 0.4 mM menadione solution in acetone (XTT:Menadione) and added to the microplate wells on which the biofilm formation on contact lenses was performed. After incubating the flat-bottom microplates for 90 min in the dark, OD readings were taken at 490 nm by using an automated microplate reader. These readings referred to the metabolic activity of the biofilms evaluated since the change in the color is proportional to the number of living cells; thus, greater the absorbance, greater is the number of metabolically active cells, considering that XTT quantifies the ability of the dehydrogenase enzyme present in the mitochondria to convert the water-soluble tetrazolium salt (XTT) (yellow color) into formazan compounds (orange color)^(8)^.

For total biomass evaluation, the wells were washed with PBS, to which 0.4% crystal violet dye was added after drying the wells/lenses. After contacting for 15 min, the dye was removed from each well containing the lenses, which were then washed 4-times with distilled water. Subsequently, absolute ethanol was added to the wells for solubilizing the dye that had adhered to the biofilm, followed by OD measurement of this solution at 595 nm. Higher OD values indicate biofilms with greater biomass production^(10)^.

The biofilm formation and assessment tests were conducted thrice, and the results were compared. Statistically significant differences (p<0.05) were then recorded and the corresponding graphs were created using the Graph Pad Prism 5.0.

## RESULTS

Both the tested *Candida* spp. could form biofilms in the evaluated contact lenses. We noted that the biofilms were formed and standardized in their growth with some aspects of cell development and adhesion.

Biofilm production by *C. albicans* SC5314 and *C. krusei* ATCC^®^ 6258 in different types of lenses was estimated by quantification of the metabolic activities by using the XTT reduction assay and analysis of fungal biomass by staining with crystal violet dye (Table 1).

**Table 1:** Metabolic activity and fungal biomass of *Candida krusei* and *Candida albicans* strains. The values are expressed as an average of the optical density (OD) reading ± standard deviation

	Candida krusei ATCC® 6258		Candida albicans SC5314	
**Lenses types**	**Metabolic activity (OD at 490 nm)**	**Fungal biomass (OD at 595 nm)**	**Metabolic activity (OD at 490 nm)**	**Fungal biomass (OD at 595 nm)**
Programmed-replacement disposal	0.380 ± 0.037	0.378 ± 0.119	0.197 ± 0.085	0.177 ± 0.045
Therapeutic	0.354 ± 0.026	0.253 ± 0.0156	0.344 ± 0.029	0.173 ± 0.048
XO^®^	0.709 ± 0.206	0.044 ± 0.001	0.722 ± 0.097	0.057 ± 0.013
Polycarbonate	0.785 ± 0.207	0.057 ± 0.007	0.694 ± 0.186	0.070 ± 0.019
Single-use	0.351 ± 0.048	0.186 ± 0.006	0.173 ± 0.062	0.295 ± 0.041

OD= optical density; XO^®^= Hexafocon A copolymer (Bausch and Lomb) lens

**Figure 1:**
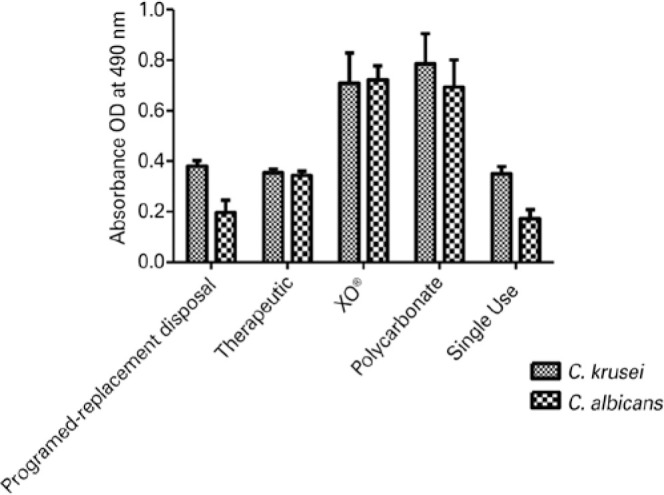
Metabolic activity of biofilms from *C. albicans* SC5314 and *C. krusei* ATCC® 6258 on different types of contact lenses.

Greater metabolic activity was determined for biofilms formed by both the *Candida* spp. on the XO^®^ contact lenses, followed by the formation in polycarbonate lenses. Biofilms with less metabolic activity were recorded for single-use lenses (Figure 1).

As shown in figure 1, comparison of the biofilms formed by the *Candida* spp. Indicated that the biofilms formed by *C. krusei* had greater metabolic activities on the programmed-replacement disposal, therapeutic, polycarbonate, and single-use lenses. However, *C. albicans* showed greater metabolic activity only in rigid XO^®^ lens.

**Figure 2:**
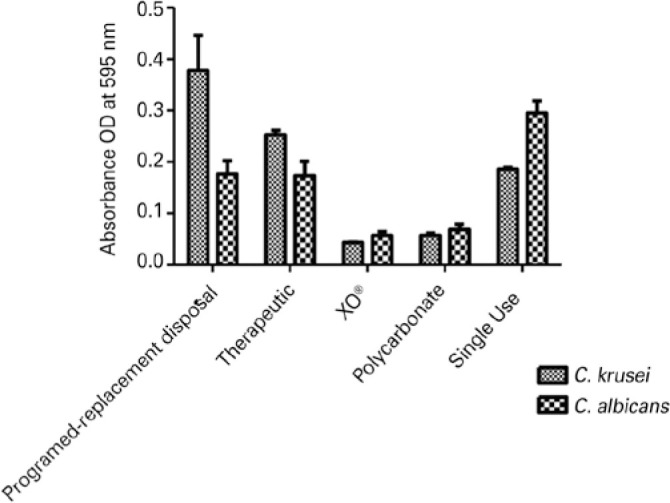
Biomass production in biofilms by *C. albicans* SC5314 and *C. krusei* ATCC® 6258 in different types of contact lenses.

Concerning the biomass analysis (Figure 2), rigid lenses (i.e., polycarbonate and XO^®^) had biofilms with less fungal biomass, although their *Candida* spp. biofilms demonstrated greater metabolic activities (Figure 1).

We also noted a large production of biomass by *C. krusei* biofilms in the programmed-replacement disposal lenses, which was approximately twice as much as that of *C. albicans* biofilms in the same type of lens (Figure 2).

In general, biofilms produced on XO^®^ and polycarbonate lenses demonstrated greater metabolic activity, but lesser representative biomass.

## DISCUSSION

*Candida* spp. constitute the normal microbiota of approximately 50% of individuals and generally reside in the human body as a commensal organism^(11)^. Several factors are associated with their virulence that guarantee their ability to colonize and cause infections, such as adherence to host cells, promotion of phenotypic changes, convergence of yeasts into pseudohyphae, formation of biofilms, production of toxic substances (such as hemolysins), resistance to hydrogen peroxide, and the production and secretion of hydrolytic enzymes^(12,13)^.

In this study, no statistically significant difference was noted in relation to the biofilm production capacity by *C. albicans* and *C. krusei* strains (p>0.05). This result contributes to the validity of the concept that contact lens can serve as a suitable surface for *Candida* spp. adhesion and growth. A past study reported that, besides that the material structure eases the adhesion and multiplication of microorganisms, corneal hypoxia resulting from the prolonged use of contact lenses tends to compromise the integrity of the epithelium, thereby acting as a gateway for microorganisms related to the causation of eye infections^(14)^.

We assumed that the lenses were not contaminated by any microorganisms, considering that the lenses arrived in sealed and sterilized packaging, which means that the results were related to the growth of sessile cells of *C. albicans* and *C. krusei* strains.

According to the results shown in figure 1, the formation of biofilms with greater metabolic activity and greater biomass was noted for rigid contact lenses (i.e., XO^®^), while the polycarbonate lenses showed greater absorbance for both the species.

We had expected that biofilms on soft contact lenses would have greater metabolic activity and greater biomass. This expectation was also related to the past reports that 2 out of every 3 infections related to the use of contact lenses were associated with soft lenses and 1 to the use of rigid ones^(1)^.

The basis for greater contamination in soft, silicone and hydrogel contact lenses, when compared to rigid lenses, can be attributed to the relative ease of removal of biofilms in rigid lenses, in addition to the fact that hydrophobic materials such as silicone with hydrogel monomers are more prone to biofilm adhesion^(15)^. This event is called superficial hydrophobicity, in which the free surface energy (FSE) contributes to the greater susceptibility to adhesion of microorganisms. This fact is also directly related to the reaction against water, which is known for its high particle adhesion capacity. Thus, greater the hydrophobicity, lower is the FSE related to the presence of water and greater is the ability of the microorganism to adhere^(16)^. In other words, hydrophobic materials, such as those mainly present in the soft contact lenses tend to enable microbial adhesion and the formation of biofilms, which is associated with higher rates of infections.

The comparison with the average values established from triplicated analyses indicated nearly similar values of metabolic activity for biofilms formed in programmed-replacement disposal, therapeutic, and single-use lenses types.

As shown in figure 1, *C. krusei* formed biofilms with greater metabolic activity in the evaluated lenses, with the exception of XO^®^ lenses; this result is consistent with that in the literature^(17)^. In a past study^(17)^, 24 *Candida* spp. were isolated (including strains of *C. albicans, C. glabrata, C. krusei*, and *C. tropicalis*). All species showed biofilm formation on acrylic surfaces with moderate to high intensity. In this study, *C. krusei* did not present with the highest values for the formation of biofilms in comparison with other species of the genus, and smaller results were presented by *C. albicans* as well; these reports conform to the present results.

Another past study indicated that more hydrophobic species, such as *C. tropicalis, C. glabrata*, and *C. krusei*, have greater ability to adhere to polymeric surfaces, such as contact lenses, while the opposite occurs with less hydrophobic species, such as *C. albicans, C stellatoidea*, and *C. parapsilosis*^(16)^.

Moreover, it can be seen from figure 2 that biofilms with greater metabolic activity do not necessarily have greater biomass. The same fact was reported in a past comparative study between *C. glabrata* and *C. krusei*, in which *C. glabrata* biofilms demonstrated greater metabolic activity, as assessed by the XTT reduction assay method, than *C. krusei* biofilms, while also producing less biomass^(18)^. As observed in previous studies, the decrease in the XTT reduction method can be directly associated with the amount of cells present in the biofilm^(18)^, while the greater metabolic activity possibly indicates greater virulence and greater resistance to antifungal agents^(19)^.

Rigid contact lenses, which are relatively more hydrophilic, are less suitable for microbial adhesion^(16)^. Among the possible causes of greater metabolic activity in these lenses with less biomass, we must consider that a greater catabolic activity, under the stress of the unfavorable environment, lead to a greater transition from organic carbon to carbon dioxide and hence greater use of oxygen from the environment. The generation of carbon dioxide results causes only a smaller amount of cells to be produced, which consequently reduces the overall biomass^(20)^. In addition, the color intensity produced in the biomass assessment test is directly related to the structure/size of the biofilm formed, which is less on surfaces that are less suitable for proliferation and formation, since, for maintaining gases and nutrients at adequate levels, large biomass condensation must be avoided^(21)^.

Generally, biofilm cells are more tolerant to antifungal treatments than planktonic cells and they can persist in the host even with a large influx of inflammatory cells and adaptive immune cells^(22,23)^. In the literature, biofilm producing *Candida* spp. have been associated with greater mortality rates when compared to biofilm non-producing strains^(23)^.

It is significant that *C. krusei* biofilms have the highest intensity of metabolic activities considering the presence of non-*albicans* strains of *Candida* spp., which are usually associated with changes in the antifungal susceptibility over a period of time; this pattern has been observed to change across the world with the expansion of the use of antifungal agents^(24)^.

Behavioral changes in a biofilm formed by the same species in relation to different topographies and substrates in terms of formation and maturation^(25)^ have been rectified by the present study. However, although our results are significant, further studies are necessary to better explain the differences between these strains. Thus, it is expected to prevent biofilm formation on contact lenses surfaces, either by manipulating their hydrophobicity/ hy drophilic relationship, by assessing the antibiofilm potential of compounds present in multipurpose solutions, and/or, mainly, by reinforcing patients to become aware of the need for correct contact lens handling.
